# Calcium/calmodulin‐dependent kinase II and memory destabilization: a new role in memory maintenance

**DOI:** 10.1111/jnc.14454

**Published:** 2018-06-27

**Authors:** Fabio Antonio Vigil, Karl Peter Giese

**Affiliations:** ^1^ Department of Cell and Integrative Physiology The University of Texas Health San Antonio 8403, Floyd Curl Drive San Antonio TX 78229 USA; ^2^ Department of Basic and Clinical Neuroscience King's College London 125 Coldharbour Lane London SE5 9NU UK

**Keywords:** calcium/calmodulin‐dependent kinase II, reconsolidation, synaptic signaling

## Abstract

In this review, we discuss the poorly explored role of calcium/calmodulin‐dependent protein kinase II (CaMKII) in memory maintenance, and its influence on memory destabilization. After a brief review on CaMKII and memory destabilization, we present critical pieces of evidence suggesting that CaMKII activity increases retrieval‐induced memory destabilization. We then proceed to propose two potential molecular pathways to explain the association between CaMKII activation and increased memory destabilization. This review will pinpoint gaps in our knowledge and discuss some ‘controversial’ observations, establishing the basis for new experiments on the role of CaMKII in memory reconsolidation. The role of CaMKII in memory destabilization is of great clinical relevance. Still, because of the lack of scientific literature on the subject, more basic science research is necessary to pursue this pathway as a clinical tool.

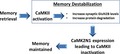

**Abbreviations used**
AIP: autocamtide‐2‐related inhibitory peptide
AMPAR: α‐amino‐3‐hydroxy‐5‐methyl‐4‐isoxazolepropionic acid receptor
CaM: calcium/calmodulin
CaMKII: calcium/calmodulin‐dependent protein kinase II
CaMKIV: CaM‐dependent protein kinase type IV
CaMKV: CaM kinase like vesicle associated
CFC: contextual fear condition
CYLD: cylindromatosis
LTD: long‐term depression
LTP: long‐term potentiation
LVGCCs: L‐type voltage‐gated calcium channels
MWM: Morris water maze
NMDAR: *N*‐methyl‐d‐aspartate receptor
PKC: protein kinase C
PKD1: protein kinase D1
PSD: post‐synaptic density
RRID: Research Resource Identifier
S‐site: substrate binding site
T286: threonine 286
T305: threonine 305
T306: threonine 306
tat: trans‐acting activator of transcription
UPS: ubiquitin proteasome system

## CaMKII

Calcium/calmodulin‐dependent kinase II (CaMKII) is the major post‐synaptic density (PSD) protein in the brain, accounting for 1–2% of total proteins (Erondu and Kennedy 1985; Peng *et al*. 2004; Cheng *et al*. 2006). CaMKII is a serine/threonine kinase composed of an auto‐inhibitory regulatory domain, an *N*‐terminal kinase domain and a *C*‐terminal self‐association domain (Chao *et al*. 2011; Hell 2014; Myers *et al*. 2017). During resting state, this enzyme is inactive because of blocking of the substrate binding site (S‐site) and the catalytic domain, both found in the kinase domain, by the pseudosubstrate segment in the regulatory domain (Braun and Schulman 1995b; Hell 2014). Binding with the calcium/calmodulin (CaM) complex causes a conformational change in CaMKII that unblocks the kinase domain from the inhibitory domain, activating the enzyme (Colbran *et al*. 1989; Meyer *et al*. 1992; Grant *et al*. 2008).

CaMKII has a wide range of substrates and is involved in many aspects of cellular function, such as the regulation of ion channel function, neurotransmitter release, gene transcription, cytoskeleton organization and intracellular calcium homeostasis (Erondu and Kennedy 1985; Tobimatsu and Fujisawa 1989; Hudmon and Schulman 2002; Lisman *et al*. 2002, 2012; Lucchesi *et al*. 2011). In mammals, four different isoforms of this enzyme are expressed: α, β, γ and δ isoforms (Tobimatsu and Fujisawa 1989; Gaertner *et al*. 2004). The most abundant isoforms in the brain are α and β CaMKII (Bennett *et al*. 1983; Tobimatsu and Fujisawa 1989; Peng *et al*. 2004). These isoforms are usually associated with each other, creating a holoenzyme composed of 12 CaMKII subunits organized into two hexameric rings (Kolodziej *et al*. 2000; Hoelz *et al*. 2003; Rosenberg *et al*. 2005). The 12 subunits are primarily composed of α and β CaMKII heteromers, but homomers consisting of only αCaMKII have been observed (Bronstein *et al*. 1988).

The enzyme is organized into a complex of subunits, thereby facilitating the occurrence of autophosphorylation. Examples of autophosphorylation sites of CaMKII are threonine 305 (T305) and threonine 306 (T306). Phosphorylation of these sites are believed to be inhibitory because of blocking of the CaM binding site, causing CaMKII to translocate out of the PSD area and decreasing long‐term potentiation (LTP) and learning (Hanson and Schulman 1992; Shen *et al*. 2000; Elgersma *et al*. 2002). The most studied autophosphorylation site of CaMKII isoforms is threonine 286 (T286) for αCaMKII and threonine 287 (T287) for βCaMKII.

Phosphorylation at the T286/287 sites occurs between neighboring subunits within the same holoenzyme, and requires binding of CaM to both of the subunits involved (Hanson *et al*. 1994; Mukherji and Soderling 1994; Rich and Schulman 1998). Phosphorylation at T286/287 allows CaMKII to remain in an active state, even in the absence of CaM, serving as an example of a CaM‐independent state of activation (Miller *et al*. 1988; Hanson *et al*. 1994; Irvine *et al*. 2006). Autophosphorylation at T286 also enhances CaM complex's binding affinity for the enzyme by 1000‐fold, with an increase in release time from less than a second to hundreds of seconds (Meyer *et al*. 1992; Tzortzopoulos and Torok 2004; Tzortzopoulos *et al*. 2004). T286/287 autophosphorylation also changes CaMKII binding affinity for other molecules. For example, it increases the holoenzyme's binding affinity to the *N*‐methyl‐d‐aspartate receptor (NMDAR) (Bayer *et al*. 2001).

Because of its ability to switch from a CaM‐dependent to a CaM‐independent state of activation by T286/287 autophosphorylation (bistability), CaMKII has been suggested to act as a memory molecule, preserving ‘memories’ of strong calcium signals (Lisman 1994). T286A‐mutant mice lack the ability to autophosphorylate at the T286 site, and have one of the most severe spatial learning deficits described in a mutant mouse (Giese *et al*. 1998; Need and Giese 2003). T286A mutation also blocks the induction of NMDAR‐dependent LTP at excitatory hippocampal CA1 synapses (Giese *et al*. 1998; Cooke *et al*. 2006).

Indeed, the role of CaMKII in learning is widely accepted; however, its role as a memory molecule is still a matter of debate (Lucchesi *et al*. 2011; Coultrap and Bayer 2012; Sanhueza and Lisman 2013). Buard *et al*. (2010) has shown that blocking CaMKII activity via systemic injection of the CaMKII inhibitor, tatCN21, prior to performing a contextual fear long‐term memory test, but after conditioning, had no effect on memory storage. Even T286A‐mutant mice can learn and maintain contextual and cued fear memory, if they were conditioned using extended protocols (Irvine *et al*. 2005, 2011). Although αCaMKII T286 autophosphorylation is required for LTP induction in pyramidal CA1 neurons (Giese *et al*. 1998; Cooke *et al*. 2006), it can also induce long‐term depression in the same cells (Marsden *et al*. 2007; Mockett *et al*. 2011) and it is not necessary for LTP induction in dentate gyrus granule cells (Cooke *et al*. 2006; Wu *et al*. 2006). Moreover, various authors have observed that LTP induction results in a transient increase in CaMKII autonomous activity, lasting for only a few minutes (Lengyel *et al*. 2004; Lee *et al*. 2009; Fujii *et al*. 2013).

Nonetheless, CaMKII has been shown to be important for memory extinction. Prolonged and repetitive re‐exposure to the conditioned stimulus without the unconditioned stimulus leads to a gradual weakening of the conditioned response, called memory extinction. Memory extinction is the learning of new environmental conditions that suppresses the previously learned conditioned response (Pavlov 1927; Eisenberg *et al*. 2003; Pedreira and Maldonado 2003; Myers and Davis 2007; Quirk and Mueller 2008; Pape and Pare 2010). The partial reduction of CaMKII autophosphorylation in heterozygous T286A mutants impairs extinction of contextual fear memory (Kimura *et al*. 2008). Furthermore, blocking of hippocampal CaMKII kinase activity impairs memory extinction (Szapiro *et al*. 2003). Inhibition of CaMKII activity by intrahippocampal injection of autocamtide‐2‐related inhibitory peptide (AIP) blocks the facilitation of memory extinction, which results from exposure to a novel stimulus (de Carvalho Myskiw *et al*. 2014). Therefore, CaMKII may play a role in memory maintenance as a biological substrate of memory extinction.

Here, we propose a different, novel and unexplored role for CaMKII in memory. We will avoid the ‘traditional’ discussion of CaMKII as a learning or memory molecule, in addition to its role in memory extinction. Instead, we will explore a different role for CaMKII in memory maintenance. CaMKII's role in memory destabilization, an important step of retrieval‐induced memory reconsolidation.

## Memory reconsolidation: destabilization and restabilization

**Figure 1:**
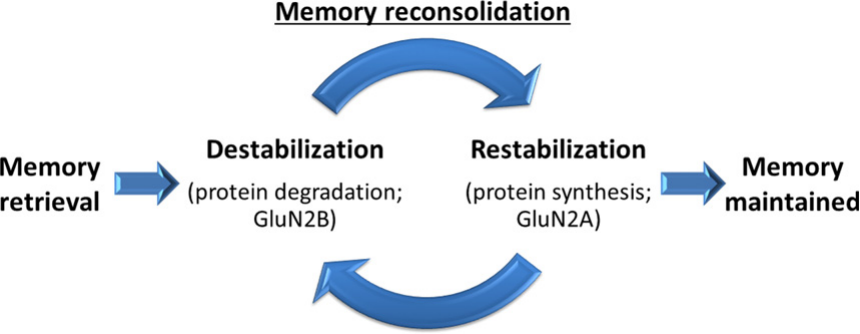
Schematic representation of how memory reconsolidation works. Retrieval of a memory by the presentation of the conditioned and/or unconditioned stimulus initiates the reconsolidation process. During reconsolidation, memory‐related proteins are degraded, which is called memory destabilization (Lee *et al*. 2004, 2008). Concurrent with reconsolidation, memory is restabilized by protein synthesis (Nader *et al*. 2000). Although it remains clear that protein synthesis is necessary to compensate for protein degradation, it is unclear if one precedes the other or if memory destabilization and restabilization happen simultaneously. Nonetheless, the result is maintenance of the memory with the possibility of alterations in the memory during the reconsolidation process (Lee 2008). Although the NMDAR is involved in memory destabilization and restabilization, it has been reported that isoform specificity can be found for these two different steps of memory reconsolidation (Milton *et al*. 2013).

The first evidence of memory reconsolidation was presented in Misanin *et al*. (1968), where an amnesic effect was induced by electroconvulsive shock 24 h after fear conditioning training. Such amnesic effect could only be achieved if the electroconvulsive shock was presented after re‐exposure to the conditioned stimulus. In other words, the associative memory between a neutral conditioned stimulus and an unconditioned stimulus was lost after electroconvulsive shock, only if the memory was retrieved (Misanin *et al*. 1968). This observation challenged the long prevailing theory that memories once consolidated would no longer be labile. Recently, memory reconsolidation has been shown to be an important process for the maintenance and further strengthening of a memory (Lee 2008; Fukushima *et al*. 2014). Retrieval‐induced reconsolidation can destabilize a memory, which involves proteasome‐dependent degradation of synaptic proteins, followed by restabilization of the memory, a protein synthesis‐dependent process (Fig. 1) (Kelly *et al*. 2003; Nader 2003; Lee *et al*. 2004, 2008; Lee 2008).

A previously consolidated memory is impaired by pharmacological blocking of protein synthesis after the retrieval process (Nader *et al*. 2000). Blocking the proteasome system with clasto‐lactacystin‐β‐lactone, a specific, cell permeable and irreversible inhibitor of the catalytic proteasome subunit 20S, reverts the memory impairment effect elicited by blocking protein synthesis (Lee *et al*. 2008). Henceforth, protein synthesis in memory reconsolidation is important to revert protein degradation‐dependent memory destabilization. The ubiquitin proteasome system (UPS) is the main mechanism for protein catabolism in mammalian cells and works by targeting proteins via ubiquitination with posterior degradation by the 26S proteasome enzyme (Varshavsky *et al*. 2000; Leestemaker and Ovaa 2017). Whether or not memory destabilization is necessary for memory maintenance after retrieval is still a matter of debate. Pharmacological blocking of the catalytic subunit 20S, immediately after retrieval, has been shown to impair memory maintenance (Artinian *et al*. 2008). However, as discussed by Artinian *et al*. (2008), it is unclear if protein degradation is solely involved in the destabilization process. The UPS might be working in memory reconsolidation by degradation of memory suppressor proteins, thereby facilitating memory restabilization. However, Lee *et al*. (2008) have reported that retrieval‐induced protein degradation by the UPS system is in fact related to memory destabilization. These authors have observed no effect on memory maintenance by pharmacological inhibition of the UPS system, after retrieval. Furthermore, Lee *et al*. (2008) have also shown that inhibition of protein degradation increases memory maintenance by inhibiting memory extinction. The apparent contradiction between the observations of Artinian *et al*. (2008) and Lee *et al*. (2008) could be a consequence of the different experimental designs. Artinian *et al*. (2008) and Lee *et al*. (2008) studies differ in terms of the hippocampal area where protein degradation was inhibited; the CA3 region was targeted in the former and CA1 region was targeted the latter. The CA1 and CA3 areas might have a different dependence for protein degradation after retrieval. Both areas have been shown to play distinct roles in memory maintenance (Ji and Maren 2008; Langston *et al*. 2010) and have different proteomic profiles (Gozal *et al*. 2002). Both articles also present results from different behavioral paradigms. While Artinian *et al*. (2008) used the Morris water maze (MWM), Lee *et al*. (2008) utilized the contextual fear conditioning paradigm. The two behavioral paradigms are known to produce different phenotypes with animals harboring the same genetic mutation (Sterneck *et al*. 1998), or even in animals exposed to the same pharmacological intervention (Shuman *et al*. 2009). The repetitive training spread throughout many days, which is required by the MWM, might create, for example, a more flexible memory that becomes more sensible to changes in the UPS function because of the continuous processes of destabilization–restabilization during training.

Nevertheless, the necessity for and the roles played by destabilization in memory maintenance after retrieval are still questions to be answered. For example, the identification of proteins targeted to degradation during the destabilization process is still poorly studied. First efforts have identified proteins involved in translational control, like MOV10 (Jarome *et al*. 2011), and synaptic structure, like Shank (Lee *et al*. 2008; Jarome *et al*. 2011). It is possible that memory destabilization plays different roles depending on the area of the brain being studied, and protein degradation could be relevant for both memory destabilization and restabilization.

## Evidences for CaMKII regulation of memory destabilization

One of the strongest pieces of evidence for the role of CaMKII in memory destabilization are the observations of Cao *et al*. (2008). By over‐expressing a transgenic form of αCaMKII that has a different ATP‐binding site structure, referred to as the αCaMKII‐F89G transgene, Cao *et al*. (2008) could increase CaMKII levels and activity, as well as specifically block αCaMKII‐F89G activity. The authors observed that if αCaMKII activity was increased at the time of retrieval of cued or contextual fear memory, the memory was specifically erased. There was no spontaneous recovery, indicating that this was a true memory erasure and not an enhancement of extinction. Cao *et al*. (2008) suggested that the memory erasure phenotype could be related to an increase in reconsolidation‐induced protein degradation, citing Lee *et al*. (2008) published in the same year.

A more direct link between CaMKII and UPS protein degradation during destabilization was the observation made in Jarome *et al*. (2016). The authors reported that administration of AIP, an inhibitor of CaMKII, in the amygdala did not affect fear memory, but it rescued retrieval‐dependent memory impairment, which was induced by blocking protein synthesis. This is a characteristic phenotype observed after blocking memory destabilization, supporting the role of CaMKII in memory destabilization. Additionally, AIP treatment stopped the retrieval‐induced proteasome activity, *in vitro* and *in vivo*, and impaired retrieval‐induced phosphorylation of the proteasome subunit Rpt6 on serine 120 in synaptosomes (Jarome *et al*. 2016). This suggested that CaMKII activity, at the time of retrieval, regulates protein degradation at the synapse.

A less substantial piece of evidence for the role of CaMKII activation inducing memory destabilization can be conjectured from the results reported by Rossetti *et al*. (2017). Rossetti *et al*. (2017) showed that viral‐induced expression of αCaMKII T286D/T305A/T306A gene (a hyperactive form of αCaMKII) in the hippocampus, blocks previously learned place‐avoidance behavior. It is plausible to propose that the expression of a highly active form of CaMKII increases memory destabilization, resulting in the memory impairment phenotype observed in Rossetti *et al*. (2017), which is in agreement with Cao *et al*. 2008;. Since the viral vector was injected 3 days after the first memory test, any CaMKII‐induced memory destabilization enhancement likely occurred during the second memory test when the phenotype was observed. However, a clear link between CaMKII activation and retrieval‐induced memory destabilization is difficult to establish because of the behavioral protocol used by Rossetti *et al*. (2017). The conditioned place‐avoidance task used demands repetitive trials to be conducted both during training and memory test, prior to viral injection. In each of these trials, memory was retrieved, which probably initiated memory destabilization/reconsolidation throughout the training and memory test sessions. Furthermore, in this behavioral paradigm, the unconditioned stimulus (shock) is present during the memory test, allowing for continued conditioning of the animal. Henceforth, this model might be too complex to deduce whether a memory impairment is retrieval dependent or independent. Rossetti *et al*. (2017) presents another plausible interpretation of the memory impairment resulting from the αCaMKII T286D/T305A/T306A mutation. Based on the memory engram theory (Tonegawa *et al*. 2015; Josselyn *et al*. 2017), the authors proposed that excessive CaMKII activity from αCaMKII T286D/T305A/T306A mutation resulted in association of the conditioned stimulus to multiple and unspecific synaptic/neuronal pathways. Consequently, the memory was lost since the memory engram was also lost. It is our opinion that the data collected by Rossetti *et al*. (2017) does not enable one to definitively determine the memory processes affected by the mutation. Even though an increase in memory destabilization or an impairment of the memory engram are possible explanations, with the data available it is impossible to determine the cause of the memory impairment.

We have recently observed that dorso‐hippocampal knockdown of CaMKII endogenous inhibitor, CaMK2N1, results in a retrieval‐dependent memory impairment (Vigil *et al*. 2017). CaMK2N1 is a specific endogenous inhibitor of CaMKII kinase activity (Chang *et al*. 1998). In our experiments, CaMK2N1 knockdown animals presented normal freezing scores in a first contextual fear memory test, but lower freezing scores in a subsequent test. This retrieval‐induced memory impairment can be interpreted as an increase in memory destabilization. We have also observed that 2 h after contextual fear memory retrieval, there was a decrease in αCaMKII T286 phosphorylation and such decrease was dependent on CaMK2N1 expression. Additionally, contextual fear memory retrieval promotes CaMK2N1 expression in dorsal hippocampi (Vigil *et al*. 2017). If CaMKII activation induces memory destabilization, CaMK2N1 expression could be induced by memory retrieval to control the destabilization process. This could explain why knockdown of CaMK2N1 results in retrieval‐dependent memory impairment. It is unlikely that this memory impairment was the result of an enhancement in memory extinction, as no extinction was observed in the control group. Extinction and reconsolidation seem to be exclusive processes (Merlo *et al*. 2014). It is important to notice that although CaMK2N1 was knocked‐down before conditioning, a memory impairment was observed only in the second memory test. This is different from Cao *et al*. (2008), who found that increased CaMKII activation impairs memory, even during the first test. This comparison leads to two important conclusions. First, memory reconsolidation is a process that extends beyond the retrieval session. Second, under physiological conditions, CaMK2N1 is important when it comes to reducing CaMKII‐induced memory destabilization after, but not during, memory retrieval. Therefore, CaMKII‐induced memory destabilization likely starts during memory retrieval, as observed by Cao *et al*. (2008), and needs to be controlled by CaMK2N1 after the memory is retrieved (Vigil *et al*. 2017).

Corroborating with a role for CaMKII in memory destabilization, Rich *et al*. (2016) performed a phosphoproteomic study of basolateral amygdala samples from rats subjected to either extinction or reconsolidation of previously learned cocaine seeking behavior. αCaMKII phosphorylation at S331, a largely understudied site, was shown to decrease when memory was retrieved and increase after extinction. In the same article, Rich *et al*. (2016) showed that S331 phosphorylation reduces αCaMKII kinase activity. Thus, reduction in S331 phosphorylation after memory retrieval is thought to increase CaMKII activity, possibly initiating CaMKII‐induced memory destabilization. If this were the case, memory retrieval would first reduce CaMKII S331 phosphorylation, resulting in memory destabilization followed by a later increase in CaMK2N1 expression in order to stop the destabilization process. Unfortunately, the authors did not test behavioral phenotypes induced by specific manipulation of S331 phosphorylation *in vivo*. Consequently, the role of CaMKII S331 phosphorylation in memory destabilization is still a hypothesis lacking substantial evidence.

The memory phenotypes of the articles cited in this section are possible observations of changes in memory destabilization by manipulation of CaMKII, and are summarized in Table 1. Based on these observations, we can raise the hypothesis that CaMKII activation during and after memory retrieval induces memory destabilization. But, it remains unclear which molecular pathways are involved in CaMKII‐induced memory destabilization. Here, we propose two possible mechanisms by which CaMKII can regulate memory destabilization.

**Table 1:** This table summarizes evidences that CaMKII activity regulates memory destabilization

Authors (year)	Journal	Brain region	CaMKII manipulation	Behavioral phenotype
Cao *et al*. (2008)	Neuron	Forebrain	Over‐expression of αCaMKII‐F86G transgene and specific inhibition of αCaMKII‐F86G kinase activity	Retrieval‐dependent erasure of tone and contextual fear memory because of over‐expression; Phenotype reversed by blocking αCaMKII‐F86G ATP‐binding site
Jarome *et al*. (2016)	Neurobiol. Learn. Mem.	Amygdala	Pharmacological blocking of CaMKII with AIP	No effect on contextual fear memory alone; Rescued retrieval‐dependent fear memory impairment induced by protein synthesis blocking, suggesting blocking of memory destabilization by CaMKII blocking
Rossetti *et al*. (2017)	Neuron	Hippocampi	Viral‐induced expression of αCaMKII T286D/T305A/T306A	Impairment of previously learned place‐avoidance memory
Vigil *et al*. (2017)	Sci. Rep.	Dorsal‐hippocampi	Viral‐induced knock‐down of CaMKII endogenous inhibitor, CaMK2N1	Retrieval‐dependent impairment of contextual fear memory

## CaMKII, memory destabilization and GluN2B

Memory destabilization was first associated with NMDAR activity by Ben Mamou *et al*. (2006). The authors show that pharmacological inhibition of NMDAR with intra‐basolateral amygdala injection of ifenprodil or AP5 prevented the retrieval‐dependent cued fear memory impairment that was induced by protein synthesis inhibition. That is, NMDAR inhibition prior to the memory retrieval session eliminated the necessity for memory restabilization, as memory destabilization was diminished.

Using more specific pharmacological tools, Milton *et al*. (2013) have suggested that within the basolateral amygdala, the regulation of destabilization and restabilization are dissociated. While memory destabilization is regulated by activation of NMDAR subunit GluN2B, memory restabilization is regulated by NMDAR subunit GluN2A. Milton *et al*. (2013) did not observe any change in auditory fear memory after specific pharmacological inhibition of GluN2B activity, but GluN2B inhibition prevented the memory impairment induced by blocking protein synthesis after memory retrieval. Hence, GluN2B inhibition impairs fear memory destabilization. In contrast, injection of a GluN2A‐prefferring antagonist, NVP‐AAM077, reduces freezing behavior after reactivation much like protein synthesis inhibition.

A GluN2B‐induced memory destabilization was also later described by Crestani *et al*. (2015) and Ferrer Monti *et al*. (2016). Both used a distractor stimulus to erase memory in a memory retrieval‐dependent matter. Crestani *et al*. (2015) used an air puff during retrieval as a distractor to induce contextual fear memory impairment. This impairment could be blocked by intra‐CA1 injection of the GluN2B antagonist ifenprodil, prior to retrieval session. Ferrer Monti *et al*. (2016) used sucrose solution after memory retrieval to erase contextual fear memory. Memory erasure was blocked by injection of ifenprodil in the basolateral amygdala. Interestingly, Ferrer Monti *et al*. (2016) confirmed that his behavioral protocol induced memory reconsolidation by using i.p. injections of midazolam, a fast‐acting enhancer of GABA‐A receptor activation that has previously been shown to disrupt memory reconsolidation (Bustos *et al*. 2006; Robinson and Franklin 2010; De Oliveira Alvares *et al*. 2013; Pineyro *et al*. 2013). Crestani *et al*. (2015), on the other hand, tested if the exposure of a rat to a different environment would induce memory erasure, and it did not, confirming the specificity and necessity of memory retrieval for memory erasure. Therefore, both articles further support the existence of a GluN2B‐induced memory destabilization mechanism.

CaMKII is known to bind to the GluN2B subunit (Strack and Colbran 1998) in a process that regulates synaptic plasticity (Barria and Malinow 2005; Zhou *et al*. 2007) and is necessary for memory formation (Zhou *et al*. 2007; Halt *et al*. 2012; Stein *et al*. 2014). CaMKII binding to NMDAR increases CaMKII activity by facilitating CaMKII T286/T287 autophosphorylation and inhibiting its dephosphorylation (Lisman and Raghavachari 2015). CaMKII complex can bind in to two different sites of GluN2B. One site is dependent on CaMKII's association with CaM (within residues 1120–1480), and the second binding site depends on CaMKII T286 phosphorylation (residues 839–1120) (Bayer *et al*. 2001, 2006). Moreover, the inhibition of CaMKII kinase activity by AIP treatment of hippocampal‐neuronal culture and hippocampal slices reduces GluN2B colocalization with PSD‐95 within the synapses (Gardoni *et al*. 2009). Hence, it is possible that CaMKII activity increases the synaptic levels of GluN2B, increasing memory destabilization after memory retrieval.

**Figure 2:**
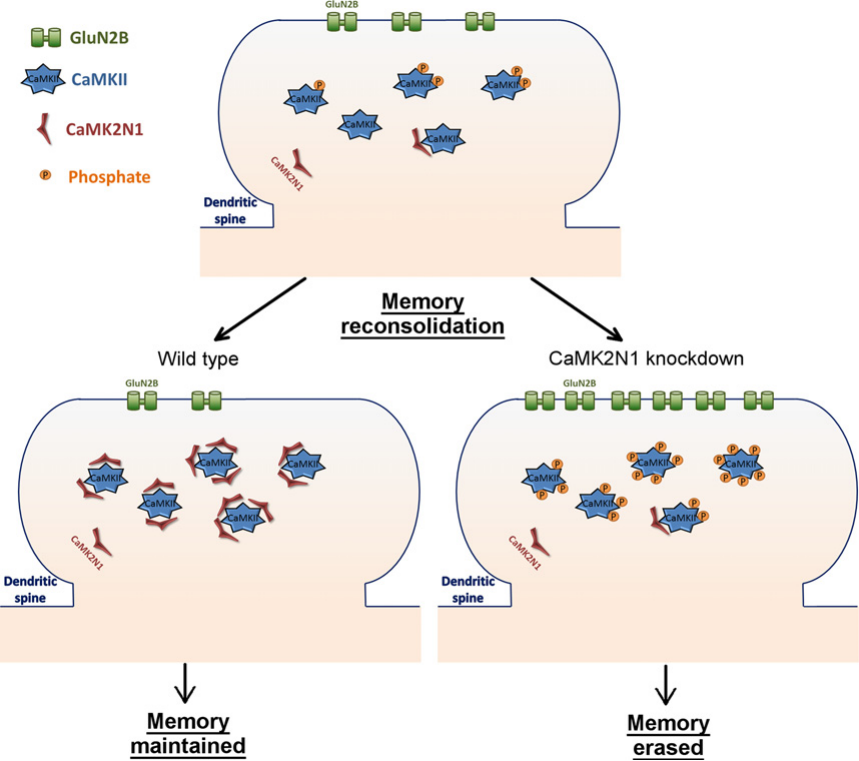
This schematic representation shows that CaMKII regulates the levels of GluN2B‐containing NMDAR in the synapse after retrieval, affecting the maintenance of a memory. This hypothesis could explain the observations of Vigil *et al*. (2017). Once memory is retrieved, CaMKII increases GluN2B localization within the synapse, starting a memory destabilization process. In normal (wild‐type) animals, this process is stopped by expression of CaMK2N1, which inhibits CaMKII and blocks the GluN2B‐induced memory destabilization. On the other hand, in Vigil *et al*. (2017), CaMK2N1 knockdown caused memory erasure because of excessive GluN2B‐induced memory destabilization resulting from the uncontrolled CaMKII activity and consequent increase in synaptic levels of GluN2B. GluN2B increase could be related to anchoring of extrasynaptic GluN2B in the post‐synaptic density (PSD) and/or decrease in GluN2B degradation. The mechanism is still unknown.

If CaMKII activity induces memory destabilization, one could predict that its inhibition is necessary for the maintenance of the memory after retrieval. In other words, excessive CaMKII activation might result in memory impairment because of excessive destabilization. Corroborating with this hypothesis, we have recently reported a contextual fear memory retrieval‐induced hippocampal expression of CaMK2N1, and this expression was necessary for memory maintenance after retrieval (Vigil *et al*. 2017). CaMK2N1 is known to block CaMKII binding to GluN2B (Vest *et al*. 2007). Thus, retrieval‐induced expression of CaMK2N1 could stop memory destabilization by blocking CaMKII interaction with GluN2B. Figure 2 is a schematic view of how CaMKII activity might induce memory destabilization via regulation of GluN2B synaptic levels and how this process would be stopped by retrieval‐induced CaMK2N1 expression. To further test this hypothesis and to understand how GluN2B activity regulates memory destabilization, more experiments are necessary.

## CaMKII, memory destabilization and protein degradation

**Figure 3:**
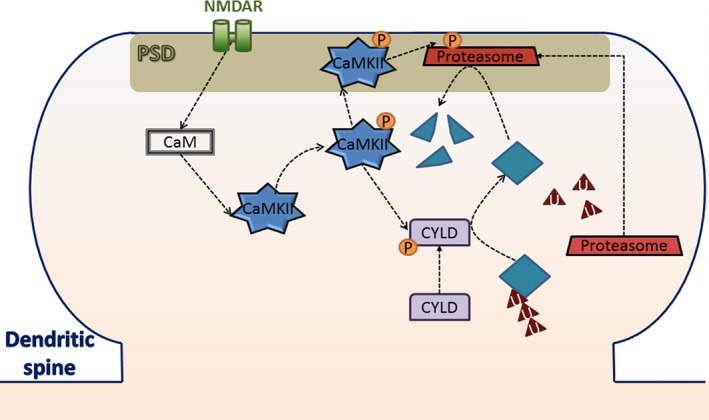
Schematic representation of how CaMKII can regulate ubiquitin proteasome system (UPS) activity and increase memory destabilization by increasing protein degradation. Calcium coming from open NMDAR binds with calmodulin creating the CaM complex that activates CaMKII. Once active, CaMKII autophosphorylates threonine 286, further increasing its activity. Active CaMKII phosphorylates cylindromatosis (CYLD), which activates this enzyme. Active CYLD removes K63‐linked polyubiquitins from proteins, targeting them for degradation. CaMKII also phosphorylates proteasome subunit Rpt6 on serine 120, increasing its activation. Active CaMKII also increases proteasome localization in the PSD. Such CaMKII/UPS pathway can help explain the behavioral phenotypes observed by Jarome *et al*. (2016) and Cao *et al*. (2008). Additionally, it is in accordance with the retrieval‐induced decrease in Shank levels, observed in Lee *et al*. (2008), and GluA1 (Vigil *et al*. 2017) levels in the post‐synaptic density.

Another possible mechanism of how CaMKII might play a role in memory destabilization is via regulation of UPS‐dependent protein degradation. It has been observed that post‐retrieval inhibition of CaMKII stops retrieval‐induced protein degradation and rescues memory impairment resulting from protein synthesis inhibition (Jarome *et al*. 2016). Autophosphorylation of T286 increases αCaMKII's affinity for the proteasome and promotes proteasome recruitment to the PSD (Bingol *et al*. 2010). CaMKII can also phosphorylate serine 120 of proteasome subunit Rpt6 and increase its activity (Djakovic *et al*. 2009; Jarome *et al*. 2013). The phosphorylation of Rpt6 seems to decrease synaptic strength by impairing miniature excitatory post‐synaptic current (Djakovic *et al*. 2012). CaMKII also phosphorylates the protein cylindromatosis. Once phosphorylated, cylindromatosis is activated and facilitates proteasomal degradation of proteins by removing K63‐linked polyubiquitins from targeted proteins (Thein *et al*. 2014). Thus, CaMKII activation increases protein degradation by incrementing proteasome activity, by anchoring it in the PSD area and by facilitating access to target proteins. This regulation of protein degradation by CaMKII is probable related to the retrieval‐induced CaMKII‐dependent proteasome activation reported by Jarome *et al*. (2016) (Figure 3). Furthermore, the retrieval‐dependent memory impairment observed after transgenic CaMKII over‐expression in Cao *et al*. (2008) experiments, could also be explained by uncontrolled activation of protein degradation. Nonetheless, a direct link between the memory maintenance impairment induced by αCaMKII‐F89G over‐expression and UPS protein degradation is still to be shown.

Although the results of Jarome *et al*. (2016) clearly support the existence of a role of CaMKII in memory destabilization by regulation of UPS protein degradation, it does not establish a definitive role for CaMKII in memory destabilization. AIP, the inhibitor of CaMKII used, belongs to a family of CaMKII inhibitory peptides designed based on T286 autophosphorylation site of αCaMKII. These peptides are fragments of the T286 area, but with a substitution of the threonine to alanine (Hanson *et al*. 1989; Braun and Schulman 1995a; Ishida *et al*. 1995; Pellicena and Schulman 2014). The specificity of such substrate‐based inhibitors of CaMKII is still a matter of debate. They have also been reported to inhibit protein kinase D1 (PKD1) (Backs *et al*. 2009) and protein kinase C (Smith *et al*. 1990; Hvalby *et al*. 1994).

A more specific inhibitor of CaMKII has been used by Naskar *et al*. (2014), the inhibitory peptide CaMKIINtide. By inhibiting CaMKII with CaMKIINtide treatment in *Lymnaea stagnalis* snail model, Naskar *et al*. (2014) described a memory consolidation impairment that could be rescued by proteasome inhibition. CaMKIINtide treatment also inhibited αCaMKII T305 autophosphorylation and decreased the levels of the α‐amino‐3‐hydroxy‐5‐methyl‐4‐isoxazolepropionic acid receptor subunit GluA1. The GluA1 decrease was rescued by proteasome inhibition (Naskar *et al*. 2014). CaMKIINtide is derived from the endogenous inhibitor CaMK2N1, and so far, it has been shown to block CaMKII kinase activity specifically (Chang *et al*. 1998; Vest *et al*. 2007). CaMK2N1‐derived peptides have also been reported to reduce levels of CaMKII at the synapse (Sanhueza *et al*. 2011), inhibit T305 autophosphorylation (Vest *et al*. 2007), block binding to Densin (Jiao *et al*. 2011) and decrease clustering of CaMKII in the dendrites (Tao‐Cheng *et al*. 2013). Although the study in Naskar *et al*. (2014) has used a more specific tool, they tested the role of CaMKII regulation for protein degradation in memory consolidation. Nevertheless, like reconsolidation, consolidation also induces a wave of UPS‐dependent protein degradation (Lopez‐Salon *et al*. 2001; Artinian *et al*. 2008; Jarome *et al*. 2011; Jarome and Helmstetter 2014). Similar mechanisms might be used in both consolidation‐ and reconsolidation‐induced protein degradation.

## Conclusion

The role of CaMKII in memory maintenance has always been a matter of debate (Lisman 1994; Irvine *et al*. 2005; Buard *et al*. 2010; Lucchesi *et al*. 2011; Sanhueza and Lisman 2013; Rossetti *et al*. 2017). Here, we have gathered pieces of evidence suggesting that CaMKII may play a role in reconsolidation‐induced memory destabilization, where CaMKII activation facilitates memory destabilization after retrieval. Cao *et al*. (2008) presents the first evidence for CaMKII‐induced memory destabilization. Jarome *et al*. (2016) established the most direct link between CaMKII and memory destabilization, identifying protein degradation as a molecular pathway involved. Vigil *et al*. (2017) supports the role of CaMK2N1 as a physiological mechanism by which CaMKII‐induced memory destabilization can be controlled. Additionally, reduction in CaMKII S331 phosphorylation could be responsible for initiating CaMKII‐induced memory destabilization (Rich *et al*. 2016). Finally, Rossetti *et al*. (2017) also observed that an increase in hippocampal CaMKII activity could lead to memory impairment. Although these observations suggest that CaMKII activation induces memory destabilization, none of these observations provides definitive evidence. Jarome *et al*. (2016) uses a pharmacological tool that is limited by its unspecific activity. Vigil *et al*. (2017) and Cao *et al*. (2008) report the occurrence of a retrieval‐induced memory erasure, but lack the direct link with a biological marker of memory destabilization. Example of these markers would be changes in S120 Rpt6 proteasome phosphorylation (Djakovic *et al*. 2009, 2012; Jarome *et al*. 2013, 2016) and decrease in the levels of MOV10 (Jarome *et al*. 2011) or in the synaptic levels of Shank (Lee 2008; Jarome *et al*. 2011). Rich *et al*. (2016) failed to study any behavioral phenotype resulting from specific manipulation of S331 phosphorylation. The retrieval dependence of the behavioral phenotype observed by Rossetti *et al*. (2017) was not tested. Consequently, experiments employing refined specific tools to manipulate and quantify memory destabilization and CaMKII activity, levels and localization are necessary.

Here, we propose two possible mechanisms by which CaMKII may regulate memory destabilization. It is possible that CaMKII controls memory destabilization via regulation of synaptic levels of GluN2B and/or via the regulation of protein degradation in the synapse. These two mechanisms can also be linked or interact with one another. The UPS activity pathway is a more direct link between CaMKII and memory destabilization, and has a larger body of evidence supporting it. The CaMKII/GluN2B pathway proposed here has never been tested and lacks the essential understanding of how GluN2B regulates memory destabilization. Aside from the involvement of Ca^2+^ influx, which is mediated by L‐type voltage‐gated calcium channels (Crestani *et al*. 2015), not much is known about the mechanism.

The hypothesis that memory destabilization is induced via a CaMKII‐dependent mechanism is not without apparent controversy. Da Silva *et al*. (2013) advocates a role for CaMKII in reconsolidation, more specifically, in the restabilization process. Da Silva *et al*. (2013) observed that hippocampal CaMKII inhibition by AIP after spatial memory retrieval induces memory impairment, which was rescued by inhibiting protein degradation. This memory impairment phenotype was time‐dependent, not present 24 h after AIP treatment but present 5 days after. So, the phenotype observed by Da Silva *et al*. (2013) was interpreted as an indication that CaMKII activity is necessary for memory restabilization. The AIP‐induced behavioral phenotype reported by Da Silva *et al*. (2013) and Jarome *et al*. (2016) are quite different from each other. However, if we consider that CaMKII might be important in both memory destabilization and restabilization, we eliminate the controversy between these two different observations. While Jarome's manipulation of CaMKII could have affected memory destabilization, Da Silva's might have changed memory restabilization. It is also important to consider that Da Silva *et al*. (2013) uses a different behavioral paradigm, the MWM. The MWM paradigm is not the most conventional paradigm used to test memory reconsolidation, as memory formation requires several training trials spread throughout various days of training. Therefore, memory destabilization and restabilization will occur during training, making it impossible to confirm the retrieval dependence of the phenotype by using a memory retrieval free group. Still, the MWM is a very useful and important paradigm to test different aspects of memory and learning.

Similar to Da Silva *et al*. (2013), Rich *et al*. (2016) also observed that intra‐basolateral amygdala inhibition of CaMKII, after memory reactivation, impaired cocaine cued memory reconsolidation. CaMKII inhibition reduced the cocaine‐seeking behavior induced by presentation of previously paired stimulus (3 tone‐light stimulation). Nevertheless, for inhibition of CaMKII, Rich *et al*. (2016) applied bilateral‐injections of KN‐93 or KN‐62, which have been shown to be non‐specific inhibitors of CaMKII. For instance, they also inhibit the kinase activity of CaM‐dependent protein kinase IV (Redondo *et al*. 2010), ‘calmodulin kinase‐like vesicle‐associated’ (Mochizuki *et al*. 1993) and others (Wayman *et al*. 2008).

It is our opinion that CaMKII likely plays a role in memory destabilization. Consequently, CaMKII plays a role in memory maintenance, not as a ‘memory molecule’, but rather as biological substrate of memory reconsolidation. If this is the case, understanding how CaMKII regulates retrieval‐induced memory destabilization could have an enormous impact on the treatment of post‐traumatic stress disorder and addiction. We also do not refute or discard the notion that CaMKII may play a role in memory maintenance via other mechanisms like extinction (Szapiro *et al*. 2003; Kimura *et al*. 2008; de Carvalho Myskiw *et al*. 2014), memory restabilization (Da Silva *et al*. 2013) or even as a memory molecule (Rossetti *et al*. 2017).

More studies are necessary to properly dissect the roles of CaMKII on memory maintenance. It is of paramount importance to include a retrieval free group in order to test the dependence of any memory phenotype to memory retrieval. The use of gene therapy treatments to knockdown, knockin or knockout specific genes can yield rich observations on the functions of CaMKII in memory maintenance. Pharmacological tools derived from the endogenous inhibitor CaMK2N1, like CaMKIINtide (Chang *et al*. 1998), are preferable because of specificity. The CaMKIINtide has been fused to the trans‐acting activator of transcription (tat) domain, increasing cell penetration and creating the 21‐amino acid peptide, tatCN21 (Vest *et al*. 2007; Buard *et al*. 2010). One can also find shorter versions like CN19 (Coultrap and Bayer 2011) and the 17‐amino acid CN17 (Gomez‐Monterrey *et al*. 2013), which have been shown to work as effectively as CN21. Transgenic animals like the T286A mutant have always been and continue to be important models for studying the roles of CaMKII in memory (Giese *et al*. 1998; Rossetti *et al*. 2017). Still, the use of inducible mutations needs to be explored further, as it avoids long‐term plasticity compensations that might bias observations. Finally, investigating the role of CaMKII in different brain areas, as well as the effect of CaMKII manipulation at different time points after the process of learning and retrieval will require a collaborative, long and challenging effort from many researchers.
